# Walking on water: revisiting the role of water in articular cartilage biomechanics in relation to tissue engineering and regenerative medicine

**DOI:** 10.1098/rsif.2022.0364

**Published:** 2022-08-03

**Authors:** Anna A. Cederlund, Richard M. Aspden

**Affiliations:** Aberdeen Centre for Arthritis and Musculoskeletal Health, University of Aberdeen, Foresterhill, Aberdeen AB25 2ZD, UK

**Keywords:** water, cartilage, collagen, aggrecan, tissue engineering, regenerative medicine

## Abstract

The importance, and the difficulty, of generating biosynthetic articular cartilage is widely recognized. Problems arise from obtaining sufficient stiffness, toughness and longevity in the material and integration of new material into existing cartilage and bone. Much work has been done on chondrocytes and tissue macromolecular components while water, which comprises the bulk of the tissue, is largely seen as a passive component; the ‘solid matrix’ is believed to be the main load-bearing element most of the time. Water is commonly seen as an inert filler whose restricted flow through the tissue is believed to be sufficient to generate the properties measured. We propose that this model should be turned on its head. Water comprises 70–80% of the matrix and has a bulk modulus considerably greater than that of cartilage. We suggest that the macromolecular components structure the water to support the loads applied. Here, we shall examine the structure and organization of the main macromolecules, collagen, aggrecan and hyaluronan, and explore how water interacts with their polyelectrolyte nature. This may inform the biosynthetic process by identifying starting points to enable developing tissue properties to guide the cells into producing the appropriate macromolecular composition and structure.

## 1. Introduction

Articular cartilage has the water content of banana, a compressive modulus comparable with that of silicone rubber, and is as impermeable as granite. It commonly provides a bearing surface with a coefficient of friction close to that of ball-bearings for the lifetime of an individual. How does it achieve this combination of properties? Answering this question would provide not only a better understanding of the function of the natural tissue and what happens when it does fail but could also lead to new avenues for developing tissue-engineered cartilage that can function successfully in the demanding loading environment of a joint.

Cartilage is an evolutionarily ancient tissue widely found throughout metazoa, being found in many invertebrates as well as vertebrates [1]. Cartilages take many forms, but all are characterized by the presence of fibrous collagens embedded in a highly hydrated extracellular matrix containing typically 60–80% water. Although water is clearly important for the transport of nutrients and removal of waste products of metabolism and catabolism, we shall concentrate here on its roles in the mechanical behaviour of the tissue. The exact composition and structural organization, however, vary not only across phyla but also, at the other end of the scale, between locations in a single species. Each cartilage is highly tuned to match the physiological requirements, largely mechanical, arising from its place in the organism. Despite their ubiquitous but diverse nature, there are fundamental ways in which cartilage composition, structure and function are inter-related to provide the required mechanical properties.

The resistance to deformation of a material, colloquially known as its stiffness, is quantified by various moduli of elasticity, depending on the nature of the forces applied: uniaxial, shear or pressure. The permeability is a measure of the ease with which fluids can flow through the material. These properties arise from the composition and internal structure of the material. For biological materials, knowledge of these properties is important for understanding the natural material and crucial for synthesizing replacement materials. Cartilage has been studied for many years and, with the rise of interest in tissue-engineered replacement materials, it has never been more important to understand the origins of its remarkable properties and how we can reproduce these biosynthetically.

The modulus of articular cartilage is in the range 0.5–100 MPa depending on the rate of loading. This enables it to support the loads generated not only by locomotion, typically several times body weight, but also impact forces during, for example, jumping, where forces up to 15 times body weight have been reported in triple jumpers [2]. It is strongly resistant to shear but is deformable enough to spread those loads over the bone, which has a modulus of approximately 10 GPa, and prevent large contact forces. However, its water content is similar to that of liver [3] and banana [4] which have moduli of about 2 kPa [5] and 20 Pa [6], respectively. One obvious difference between cartilage and banana is that water is not easily squeezed out of cartilage. The hydraulic permeability of the tissue is comparable with that of granite, one of the most impermeable of rocks. If water is unconstrained it has virtually no resistance to shear and flows under gravitational forces; if it is contained and compressed, it has a bulk modulus of about 2 GPa, approximately two to three orders of magnitude larger than that of cartilage. If water is so constrained in cartilage by low permeability, why then is the modulus so much lower than that of constrained water yet so much greater than that of banana? Is water simply an inert filler that is squeezed out through tiny pores or does it play a structural part in the load-bearing capability of the tissue?

There have been numerous models for the mechanical functioning of articular cartilage, each highlighting a different aspect of the composition or structure and having more or less resemblance to the physical reality of the tissue. Perhaps the most prevalent concept is that of a water-filled solid in which the solid component carries the load while the water flow determines the time-dependent nature of the deformation under load. This is similar to common models of soils, called consolidation, a description of which was formulated by Biot [7]. Soils, however, have a typical water content of 0.2–0.5 v/v, much lower than that of cartilage. In articular cartilage, a common oversimplification is that the collagen provides the tissue with its tensile properties and the aggrecan those in compression. Like any composite material, however, the properties in compression and in tension are dependent on both these components, on interactions between them and, crucially, the water they contain.

In tissue engineering, just as in traditional engineering, understanding the origins of a material's properties is fundamental to adapting it for structural use or designing more appropriate synthetic materials [8]. In a recent Fell-Muir lecture summarizing our current understanding of cartilage, Hardingham emphasized a need for progress, ‘… in the particular challenges of understanding how molecular properties can explain tissue macro properties’ [9]. While a reductionist approach to identify fundamental factors and causal relationships is a legitimate and common approach, the constructionist phase is more problematic because properties can emerge in ways that are not always easily predictable [10]. Here we review the composition, structure and properties of articular cartilage and propose that water is an integral part of this structure with a vital load-bearing function. By orienting our approach around the role of water, the main constituent, rather than solely the macromolecular components, we hope to cast new light on relationships between composition, structure and function. The fluidity of water is essential to generate the appropriate time-dependent mechanical stiffness that enables the tissue to cope with impact forces arising from jumping as well as prolonged periods of standing. A better understanding of how articular cartilage does this could lead to new avenues for developing tissue-engineered cartilage that can successfully sustain the demanding loading environment in which it has to work. A biosynthetic approach has potentially a crucial role in regenerative medicine for damaged synovial joints, but one of the main challenges is that of generating tissue with sufficient mechanical strength, stiffness and toughness to implant successfully into a functioning joint.

## 2. Composition

The molecular composition of articular cartilage has been studied in increasing detail as technology has improved. The ability to dissolve the tissue in 4 M guanidine hydrochloride [11] and advances in gel chromatography, electrophoresis and density-gradient centrifugation technology enabled the basic composition to be established as collagen type II, aggrecan (formerly called large aggregating proteoglycan), hyaluronan (HA) (hyaluronic acid) and link protein (summarized in [12]). Muir [12] opens, however, by pointing out that the water content is more than 65% and up to 80% near the joint surface and that this is surprising for such a ‘strong’ (*sic*) tissue. Fessler [13] experimented with mixtures of HA and collagen and commented that, ‘Water … would form the basis of a structure withstanding compression, provided that its free flow out of a structure which is being squeezed is hindered’. Interestingly, since that time the focus has been almost exclusively on the second half of his statement, how the flow of water is hindered, rather than how water might form the basis of a structure; a subtle but important distinction.

The number of proteins and proteoglycans in cartilage that have been identified has expanded considerably since those early studies and the repertoire now includes numerous small leucine-rich proteoglycans (SLRPs) [14,15] and members of the matrilin and thrombospondin families (reviewed by Heinegård [16]). Many of these interact with fibrillar collagen, collagen type VI and with cell-surface molecules, thereby providing opportunities not only for linking within the extracellular matrix but also between the matrix and the chondrocyte. Diffraction studies of decorin, one of the SLRPs, have indicated a ‘banana-shaped’ structure [17] and although it crystallized as a dimer it is reported to be a monomer in its biologically active form [18]. SLRPs bind to collagen, some of them by more than one binding site, and a major function is the assembly of collagen fibrils and the collagen matrix [14]. Whether they regulate collagen cross-linking or bridge between fibrillar and short-chain FACIT collagens (fibril-associated collagens with interrupted triple-helices) remains to be determined [14]. Despite a growing wealth of knowledge about the structure and binding properties of SLRPs and other proteins and proteoglycans, and the effects of selective inactivation by genetic manipulation [19,20], their actual function within the matrix remains largely unknown.

That interactions, not just entrapment, between collagen and proteoglycans are important was indicated by studies showing that changes in the conformation of collagen preceding denaturation are necessary before proteoglycans are released [21]. Further evidence for inter-molecular interactions came from X-ray diffraction studies, measuring alterations in layer-line intensities in diffraction patterns on reducing the proteoglycan content [22], and, more recently, from measurements of interaction forces by atomic force microscopy [23]. X-ray diffraction [22] and electron microscopy [24] indicate that proteoglycans are regularly ordered on the type II collagen fibrils and so interact specifically with certain combinations of amino acids found at the fibrillar surface.

### 2.1. Water

Water itself has been a surprisingly controversial component of cartilage, and arguments raged for many years over how much, if any, was ‘free’ or ‘bound’. Because of the structure of water, with its large dipole and free electron pairs (discussed below), a fraction of the water was believed to be ‘stuck’ to the proteins and proteoglycans and be unavailable for solvation or flow. Maroudas and Schneiderman showed, however, using tritiated water as a tracer, that almost all of the water is freely exchangeable, both extra- and intra-fibrillar [25]. Some of the intra-fibrillar water is required for collagen triple-helix formation by providing water bridges between the alpha-chains and thereby contributing to the stability of the collagen triple-helix. This does not imply, however, that these water molecules are ‘bound’ in a static configuration and even these appear to be exchangeable. Intra-fibrillar water, though, is not accessible to solutes or to proteoglycans. Maroudas *et al.* have pointed out that the effective concentration of proteoglycans is that found in the extrafibrillar space and that calculated ionic partition data based on total tissue water can be in error by more than 100% [26].

The structure of water itself continues to be a puzzle and its coordination with ions and macromolecules adds another level of complexity. Pure water shows a tetrahedral coordination and the mean separation of water molecules is about 0.45 nm [27,28]. The addition of metal ions, for example sodium, leads to an octahedral hydration pattern with a mean Na–O distance of 0.234 nm in the first coordination shell, which in turn pulls the second shell closer so that the mean separation of water molecules within this hydration shell becomes approximately 0.33 nm [29]. A similar compression of water has been observed in model systems using gly-pro and ala-pro dimers [30], which have similarities to the collagen primary structure (gly-X-Y) (see below). Interestingly, the opposite effect, a reduction in density, and the proposal that water sustains a tensile stress up to 100 MPa, has been reported in hydrophilic confinement such as may be found in reverse micelles [31]. While this is a very complicated area and still subject to investigation, these studies indicate that water itself can be strongly affected by the local environment and may form the basis of a structure capable of sustaining considerable stress, as was suggested by Fessler [13]. This will be explored in more detail in relation to collagen and aggrecan.

### 2.2. Collagen

Collagen fibrils are the principal constituents of connective tissues as diverse as bone, cartilage and meniscus, which primarily support compression, to ligaments and tendons that transmit uniaxial tension. In between are numerous structures that form pressure vessels (blood vessels and intervertebral disc) or contain and constrain other tissues [32] (such as skin and fascia). In all these tissues, the rope-like collagen is organized in such a way as to be placed into tension during loading [33]. The major collagen in most of these tissues is type I collagen; articular cartilage is one of the few tissues that contains primarily type II collagen (along with nucleus pulposus and costal, tracheal and nasal cartilages). The reasons for this are not known. The molecular organization of collagen has been known in general terms since the pioneering work of Ramachandran *et al.* [34,35], and recent reviews present more detail than is warranted here [36–38].

Most studies of collagen structure have used type I collagen and much less is known about collagen type II [38]. Although the collagen type I molecule is a heterotrimer, comprising two *α*1(I) chains and one α2(I) chain, and collagen type II is a homotrimer (α1(II)), these alpha-chains have very similar primary sequences and might reasonably be expected to have a broadly similar rope-like conformation. Type II collagen is characterized by the same triplet of amino acids, typically Gly-X-Y [39] where the amino acids in the X and Y positions are often (2S)-proline (Pro, 28%) and (2S,4R)-4-hydroxyproline (Hyp, 38%), respectively [40] in the triple-helical domain of 1014 residues. The fundamental molecular structure is a right-handed helix comprising three left-handed helical chains. Proline and hydroxyproline together comprise 22% of all amino acids in most fibrillar collagens [41]. Proline is amphiphilic, able to interact with water and lipids, and it interacts strongly with water in its immediate environment but in such a way that does not disturb the bulk water structure [42,43]. Molecular dynamics simulations indicate between 19 and 25 water molecules for each proline in solution independent of temperature [43,44]. As a result, the tetrahedral structure of water remains intact, even though the polar portions of the proline molecule are sufficiently hydrated to ensure its high solubility [42].

Collagen molecules are about 300 nm long and their axial arrangement, with a 234-amino acid pseudo-period, is largely uncontested. When aggregated into fibrils they give rise to a 67 nm repeating pattern that has been observed using a variety of physical imaging methods. Collagen fibrils, however, seem never to exist in pure form and type II collagen in hyaline cartilage is co-polymerized with collagens type XI and type IX [45]. Reasons for the heterotypic nature of the fibrils may be to fine-tune the properties; type XI collagen forms a filamentous template at the core of the fibril and regulates fibril diameter through its retained N-propeptide domain [46]. Fibril diameters are about 30–80 nm, with coarser fibres being more common in the deeper zone of articular cartilage, and from many scanning electron microscopy studies, it appears that fibrils are separated by distances of the order of 40–400 nm [47].

Molecules of type IX collagen [48] lie on the surface of type II fibrils in an anti-parallel arrangement [45]. The molecule comprises four non-collagenous domains (NC1–4) interspersed by three collagenous (triple-helical) domains. NC4 and one of the collagenous domains project from the molecular surface and have been proposed to be a source of interactions between fibrils enabling collagen to make up a ‘framework’ within the tissue. Type IX was hypothesized to ‘glue’ together type II fibres, possibly via the interaction of two NC4 domains on different fibrils with a common PG [49], although this has not yet been proven.

Because of its extended helical structure and the large number of proline and hydroxyproline residues at the fibril surface, collagen has a strong ordering effect on the surrounding water. X-ray diffraction studies on collagen-like polypeptides demonstrate that polyproline II triple-helices, a collagen-like structure, are surrounded by a highly structured cylinder of hydration that determines their lateral separation in macromolecular assemblies [50]. Three water environments have been proposed to provide a sheath of water surrounding collagen, and electron microscopy of vitrified samples has provided visual evidence for this [51]. Essential to the triple-helical structure of collagen is a water bridge between a positive amide group and an adjacent negative carbonyl for every three amino acids [52]. This accounts for 0.0658 g water per g collagen. Berendsen [53] proposed an additional three water molecules per tripeptide unit, corresponding to 0.197 g water g^−1^, residing in the grooves between each pair of peptide chains. Together with the water bridge, this forms a chain of four water molecules per tripeptide repeat. He concluded, ‘Thus the collagen macromolecules may stabilize the existence of chain-like structures in the water of hydration by the formation of hydrogen bonds at appropriate sites' [53]; an unexpected way of viewing the relationship between collagen and water where normally we think of water stabilizing the collagen. The total water content of collagen is about 1.62 g water g^−1^ collagen, and the remaining 1.315 g water g^−1^ is reported to be in a monolayer at higher energy relative to the first two compartments but still lower than bulk water [54]. Although these three water compartments in turn might appear to be more tightly ‘bound’, nuclear magnetic resonance shows that rapid exchange leads to them being indistinguishable as a weighted average of the three states [55], thus finding agreement with Maroudas regarding their freely exchangeable nature [25]. Interestingly, although aggrecan is largely credited with attracting and retaining water in the tissue, the mass fraction of water based on collagen alone is, therefore, about 15% w/w of the tissue.

### 2.3. Aggrecan

Aggrecan in human articular cartilage has a protein core with a molecular weight of 245 kDa and is richly decorated in a very characteristic pattern with long-chain glycosaminoglycans (GAG) resulting in a total molecular weight of about 2–4 MDa. The detailed structure, with its three globular domains (G1–G3) and the keratan sulphate (KS)-rich and chondroitin sulphate (CS)-rich (CS1 and CS2) domains, has been described many times [16,56–59]. While its ability to retain water and its interaction with HA and link protein at the G1 domain are widely recognized, aspects of its structure that enable it to interact with other matrix macromolecules are less well studied. Unlike collagen, there is considerable variation in aggrecan both between and within species. Both the KS and the CS domains are variable between species and length polymorphism, as well as variations in CS chain length and sulphation have been reported in the CS1 domain in humans [56]. The contour length of the protein core has been reported to be about 330 nm from bovine and porcine tissues using electron microscopy [58] and slightly longer, up to about 400 nm, in human tissue using atomic force microscopy [60]. Before considering in more detail the conformation of the core protein, some more detail is required of the associated GAGs.

There are about 100 CS chains attached to ser-gly recognition sequences and 20–50 KS chains, mainly O-linked to serine and threonine, along the protein backbone [16,61]. KS chains have an extended form and diffraction studies indicate this is a twofold helix with an axial rise per disaccharide of 0.945 nm [62]. The molecular weights of adult human aggrecan-related KS chains were reported to be between 5700 and 8250 Da [63] implying 14–20 disaccharide units and, hence, an extended chain length of approximately 13–19 nm. Most of the monosaccharides are reported to be sulphated. The CS chains range from 10 to 30 kDa, equivalent to about 20–60 disaccharide units, depending on species and age, corresponding to lengths of about 20–60 nm in a variety of tissues [58,60]. CS chains are reported to be shorter in the CS2 region than in CS1 [56]. X-ray diffraction of CS has found either two-, three- or, less likely, eightfold helical conformations [62,64], depending in part on the nature of the cation environment [65]. The axial rise per disaccharide is, again, about 0.95 nm [64] and the suggested threefold helix results in an angle of 120° per disaccharide around the axis of the chain [66]. Measurements using atomic force or electron microscopy support these dimensions [58,60]. The extended helical conformations result in charged sulphate groups being well-distributed around the helical axis and the result is that a mole of aggrecan contains about 4500 moles of sulphate and 4200 moles of carboxyl groups [61]. Such a high charge density will try to extend the molecule and the traditional ‘bottle-brush-like’ structure in solution leads to a cylindrical conformation with a diameter of about 80–100 nm and a length of 350–400 nm. This is supported by electron microscopy of spread proteoglycan aggregates after rotary shadowing, showing that the CS-rich region is clearly separated from the central filament of HA [67]. There is, currently, no evidence it maintains this conformation in cartilage.

**Figure 1.**
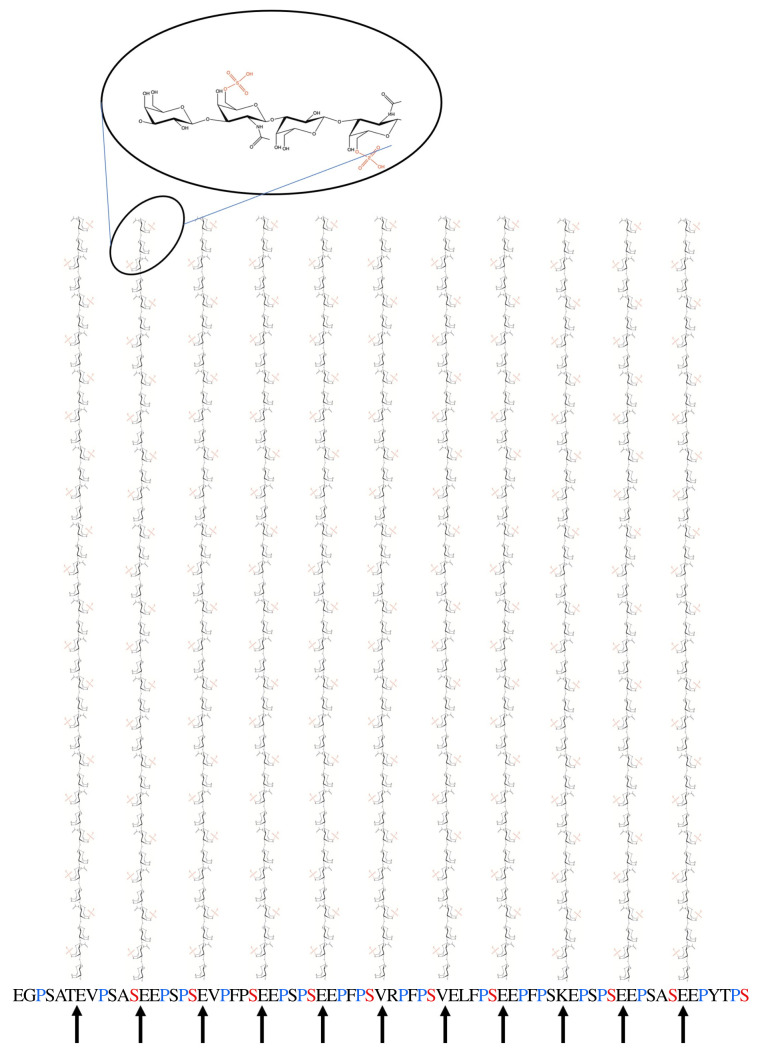
Eleven hexapeptide repeats in the aggrecan sequence [70] (starting with glutamic acid (E)) are proposed to form an extended polyproline-II-like (threefold) helix. Each hexamer is then approximately 1.86 nm long. This indicates that the KS-chains, which form a twofold helix, all project on one side and their sulphates on alternate sides form a highly anionic plane of dimensions approximately 20 × 20 nm. KS-chain diagrams are adapted from diagrams published CC-BY by Selberg *et al*. [73].

Returning to the core protein, the G1 and G2 globular domains are separated by a relatively rigid spacing sequence, E1, of about 25 nm resulting in a G1–G2 spacing of about 21 nm [58]. Following G2, there is the KS-rich domain comprising a repeated highly conserved hexapeptide motif with between four repeats in rat [68] to 23 repeats in bovines [69], with humans being one of a number of species having 11 repeats [70]. Each hexapeptide contains at least one and commonly two proline residues and comparison with collagen suggests that this region will adopt a polyproline II-like conformation, for which there is some experimental evidence (Cleis R. 1989 *Polyproline II helix as a secondary structure element in proteins* (Personal communication) Biozentrum der Universität in Basel). The result is that it will form a left-handed helix with threefold symmetry and three residues (0.93 nm) per turn [71]. The whole KS-rich region, then, is about 20 nm long in humans with each hexapeptide containing one KS chain O-linked to a serine or threonine [72]. The serines, which appear every sixth peptide, will all lie on the same side of the helix, meaning that the KS chains attached to these serine residues all lie in a plane, separated by about 1.86 nm, and all pointing in the same direction (figure 1). In addition, the twofold helix for KS described above suggests that the sulphates project sideways, 180° apart. This potentially presents a large planar, highly charged area, approximately 20 by 20 nm, for possible interactions with water and macromolecules such as collagen. In support of this, the KS-rich region isolated from aggrecan by trypsin digestion has been shown to bind with high affinity to collagen [74]. Interestingly, using immunogold technology, this study also showed that this particular domain is co-localized in the tissue with the so-called gap regions of the collagen fibres, particularly in the pericellular and territorial matrix of cartilage.

**Figure 2.**
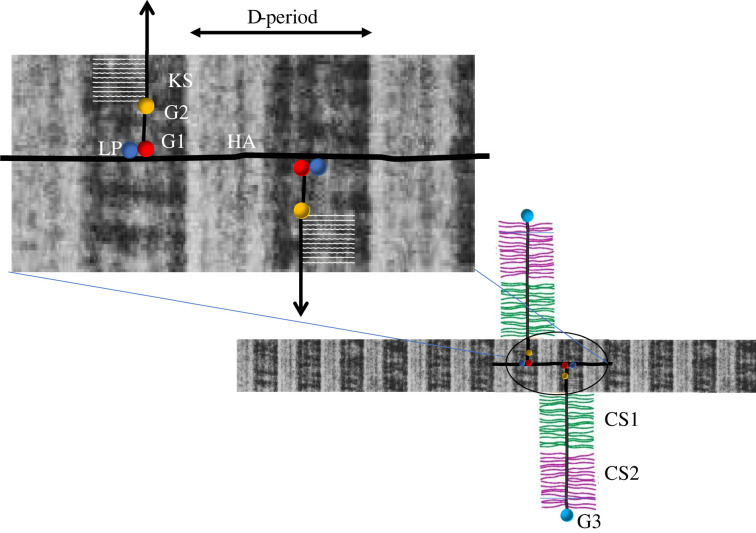
Schematic diagram, approximately to scale, showing hypothesized relationships between the hyaluronan-aggrecan complex and collagen fibrils. Aggrecans associate via their G1 globular domain along the HA molecule stabilized by LP. Assuming there is rotational flexibility, they can lie either side of the HA. This assembly then can lie along a portion of the collagen fibril leaving a gap between CS-regions of adjacent aggrecans of approximately the same dimensions as the fibril diameter. The KS-region of the aggrecan is localized to the gap region of the collagen and the KS chains all point in the same direction. The CS-regions (CS1 and CS2) then protrude into the inter-fibrillar space and have been shown extended although their conformation is unknown.

Many schematic diagrams of cartilage show random or stylized depictions of collagen and the HA-aggrecan complex, generally not to scale. Exceptions show HA lying alongside the collagen, deduced from its ability to protect the collagen from enzymic degradation [75], or even lying over the collagen [76]. From the above analysis, it appears that the KS-rich region acts as a spacer within the proteoglycan aggregate, adding to the G1–G2 spacing described above. Combining the dimensions of these regions, we have 21 nm G1–G2 plus 20 nm for the KS region before the start of the CS1 region. If there is torsional flexibility in the HA molecule then it is feasible that the aggrecan molecules could extend on both sides of the HA core leading to about 80 nm between CS1 regions. Is it coincidence that the dimensions of this region are similar to the diameter of a type II collagen fibril [77]? Could the KS-regions then ‘clasp’ the collagen leaving the CS regions more extended, similar to that portrayed by Reginato and Olsen [76] (figure 2)?

CS chains attach to serine-glycine recognition sites and are found in two identifiable domains CS1 and CS2. CS1 comprises 29 19-residue repeats, each ending with two ser-gly repeats separated by leu-pro. The CS2 domain shows no apparent order and has 69 ser-gly pairs out of 665 residues. The CS regions have no sequence similarity with any existing protein and are generally believed to be intrinsically unstructured, although gaps between areas that are substituted and the requirement to occupy a smaller volume in cartilage than in free solution suggest that it may be folded in some way. In an attempt to study this, an unglycosylated 30 kDa peptide from the C-terminal CS2 region was produced and, using a variety of biophysical techniques, shown to form a stiffened and elongated semi-ordered structure [78]. The existence of secondary structure in the form of a segmentally flexible chain was described as ‘wormlike’. Using electron microscopy and comparison with images of fully glycosylated aggrecan molecules, it appeared that glycosylation resulted in further extension of the chain by about 25% [78]. Aggrecan monomer and aggregate showed surprising differences in their Raman water peaks. The intensity relative to the CH peak was very much higher in the aggregate than in the monomer. Whether this arises from interactions with the binding region components is an important question for the future, because it suggests that interactions with water may be a significant factor during the formation of aggregates [79].

### 2.4. Hyaluronan

Up to about 100 aggrecan molecules bind to HA, a non-sulphated acidic GAG, to produce a huge macromolecular complex of over 200 MDa [80]. The G1 domain of aggrecan was found to bind to a specific decasaccharide segment of HA [81]. The interaction is stabilized by a small glycoprotein called link protein, which has affinity for both HA and the G1 domain of aggrecan [82]. Later studies indicated that the combination of the G1 domain and link protein interacted with about 20–25 disaccharides and that when either G1 or link protein alone was added in sufficient amounts to saturate HA binding the length of the HA, normally about 1 nm per disaccharide in extended conformation [83], was reduced to half its length [84]. Addition of both to form a ternary complex stabilized the aggregates and resulted in the dense heavily stained structure seen under the electron microscope with aggrecans spaced about 12 nm apart along the HA backbone [84]. Later studies, using newly synthesized matrix from chondrocyte culture, have reported the spacing between aggrecan attachment points to be 26–27 nm [85], suggesting no contraction of HA. The discrepancy was attributed to a possible difference between extracted and re-associated complexes. They did show, however, that younger donors could synthesize HA chains that could carry over 200 aggrecan molecules and this chain length declined with the age of the donor [85].

To summarize this section, an appreciation of the composition leads to a better understanding of the organization and function of the tissue and the crucial role of water within this. To replicate this, it is crucial for cells to express an appropriate phenotype. Even though we have dealt only with the key components of cartilage, it is clear that proper ratios and abilities to interact are crucial to developing the structures seen in the natural tissue. It has been shown previously that tissue elasticity determines cell lineage and commitment [86], and more recently the fate of MSCs was found to be dependent on the stiffness of the extracellular matrix and independent of protein tethering [87]. Failing to start this feedback loop in the right place with cell phenotype and matrix stiffness in step, or any attempt to short-cut this in tissue-engineered materials using a synthetic matrix, may be one reason why it is proving difficult to develop the required mechanical properties for the tissue.

## 3. Molecular organization

**Figure 3.**
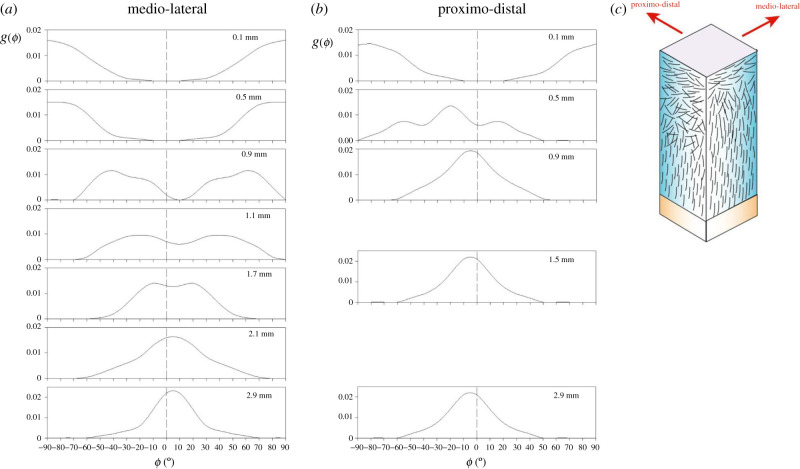
Measurements, using X-ray diffraction, of the preferred orientation and orientation distribution function as a function of depth from the articular surface in human patellar cartilage. There are differences between views in (*a*) a medio-lateral direction and (*b*) a proximo-distal direction resulting in different apparent thicknesses of the surface, transition and deep zones. This is shown schematically in (*c*).

Having established some foundations related to the composition of cartilage, the organization of those components is equally crucial to its ability to structure water and, hence, its mechanical function, and this will be considered next. Although commonly modelled as a uniform material, there is a well-established zonal variation within the tissue. Composition, collagen organization and chondrocyte morphology all vary with distance from the articular surface towards the subchondral bone. Consequently, the tissue does not possess a unique set of material properties; it is anisotropic and inhomogeneous, which makes mechanical testing and material characterization challenging. The collagen organization has been described in terms of arcades in human tissue [88], leaves in bovine [89] or even tubular structures in rabbit cartilage [90]. All this indicates variations on a theme whereby the collagen organization resists the internal expansion produced by the proteoglycans while anchoring the tissue to the bone. Exactly how it does this, however, tends to be assumed rather than proven. Most descriptions of the arrangement of collagen are qualitative, but X-ray diffraction [91,92] and polarized light microscopy [93–95] have yielded quantitative descriptions of the collagen organization. In human tissue, these largely confirmed Benninghoff's arcade model [88] albeit with distributions that varied slightly depending on the location in the tissue and the direction of observation (figure 3). The nature of the surface of cartilage has proved controversial but is clearly important for lubrication and movement. The zonal structure is laid down during embryonic development and, with a negligibly slow collagen turnover in the adult tissue, appears to fix the integrity of the tissue throughout the lifetime of the organism. Activation of collagenases in disease destroys the tissue and repair seems, at best, to be a plug of fibrocartilage. Intriguingly, intrinsic repair of a small cartilage defect has been found in some strains of mice [96] leading to hopes that we may be able to adapt this for a therapeutic approach in humans.

### 3.1. Surface zone

Surfaces of any material are difficult to study, as their surface energy leads to structural modifications and molecular adsorption from the surroundings. Cartilage is no exception, and there have been many debates over the detailed composition and structure of the articular surface. Some things, however, are clear. There is no surface membrane or lamina. Such a delicate structure could not survive the mechanical environment, and the lamina splendens was clearly an artefact of the technique (phase contrast microscopy) [97]. The coefficient of friction is remarkably low and has been extensively studied (see review by Klein [98]) with reported values in the range 0.001 to about 0.01. This compares with about 0.07 for PTFE (Teflon), one of the lowest of any known solids, and about 0.02 for mixed lubrication of ice. Synovial fluid alone may not be an exceptionally good lubricant; friction coefficients around 0.02 have been reported for cartilage on glass lubricated by synovial fluid [99]. A key factor appears to be a glycoprotein called Proteoglycan 4 (PRG4) previously known as lubricin [100] or surface zone protein expressed within the cartilage. Recently, a mechanism has been proposed of negatively charged polymers (aggrecan and PRG4) forming a brush-like zone at the surface and attracting counter-ions. These trapped ions, together with the highly polarizable nature of water molecules, form a hydration sheath between the surfaces [98,101]. Although this would explain the lack of ‘stick-slip’ on the initiation of movement, recent studies suggest this mechanism still cannot fully explain the remarkably low values for the coefficient of friction [102]. Adsorbed phospholipids have been identified on the surface of articular cartilage with phosphatidylcholine being the most common head-group and oleic acid (C18:1) the most abundant associated fatty acid [103]. This has led to a proposal that the extreme reduction of friction in aqueous media is due to close-packed layers of phosphatidylcholine vesicles which, combined with the zwitterionic polymer brushes, enable lubrication by hydration shells that surround, and are attached to, charges in water [104]. These are highly fluid and effectively act like ball bearings between the surfaces even at the high stresses (typically approximately 10 MPa) found within a joint.

The outermost surface layer of articular cartilage is reported to be acellular and non-fibrous and is up to about 1 µm thick [105], which is greater than the characteristic chain-lengths of most macromolecules. It would seem most likely that the protein and proteoglycan components are released by the surface zone chondrocytes and migrate to the interface [98], but the origin of the adsorbed phospholipids is currently unknown. Raman scattering has demonstrated the presence of lipid in the pericellular matrix of superficial zone chondrocytes [106], which may indicate that this could be the source rather than the synovial fluid. It follows that superficial zone chondrocytes in engineered tissues may need to express a different phenotype to those in the deep zone in order to synthesize the appropriate molecules to maintain the surface integrity of implanted cartilage. It is clearly important to ensure effective lubrication to avoid the risk of high frictional forces rapidly destroying any freshly implanted material.

### 3.2. Deeper zones

The organization of the collagen fibrils changes from an alignment primarily parallel with the articular surface within the surface zone to one perpendicular to the surface, and to the bone, in the deep zone. In between is a transition zone in which collagen has a bimodal distribution (figure 3). Human cartilage from the femoral head is much thicker than that from bovine femoral heads [95], very divergent from the positive correlation with body size found between bovine and other quadrupeds [107]. Using polarized light microscopy, the surface and transition zones have also been shown to be much thicker in human cartilage than in bovine [95], which were more similar to those found in pig [93] and dog [94]. It was suggested that these thicker zones might explain why the tissue is more resistant to cracking and better at sustaining impact loads than bovine tissue [95]. Most animals used as models for studies of cartilage rarely jump and subject their tissues to impact loads. In summary, the subtle variations in structure appear to be carefully crafted to adapt each tissue for its particular load-bearing function, presenting fresh challenges for tissue regeneration.

## 4. Material properties

Water is often treated as an inert fluid that saturates a solid cartilage matrix, and it is commonly assumed that the properties of the solid, its modulus, permeability and porosity determine the properties of the tissue. Having established some salient features of the matrix macromolecules, we will examine this assumption to provide further evidence that water itself is a key load-bearing component.

### 4.1. Permeability and fluid loss

Treating cartilage as a fluid-saturated solid means that any applied load will create a volume change that is due to loss of fluid. This will be especially true if both are considered incompressible as the volume of fluid lost must then be equal to the volume change in the tissue during compression. The question then arises, how much fluid is expressed and what are the new volume fractions of fluid and solid? In early studies, fluid loss due to loading was found to be up to about 30% of the tissue mass, depending on the load and for how long it was applied [108,109], although the tissue weight but not its volume was measured. The rate of loading, however, has rarely been considered until recently. Milentijevic & Torzilli [110] claimed to be the first to measure fluid loss during rapid loading. Cartilage samples were loaded in confined compression through the base with the surface pressed against a stainless-steel porous filter and strains of up to 25% recorded [110]. They noted that water loss was always less than the volume loss due to the applied strain and did not exceed 15% of the total water content. Water loss decreased with shorter loading times and by extrapolating to zero time they predicted a loss of about 2% would still occur during ‘instantaneous' loading. By contrast, studies using a drop tower to apply impact loads that produced strains in unconfined compression of up to about 50% have reported water loss was undetectable to within 0.1% [111]. Later studies found values for Poisson's ratio very different from that for an isovolumetric deformation but still no indication of fluid loss [112].

Water retention within the tissue is attributed to an extremely low permeability and a low value was believed to be essential for the self-lubricating properties of the tissue. Early measurements produced a value of 5.8 × 10^−13^ cm^3^ s g^−1^ (5.8 × 10^−16^ m^3^ s kg^−1^ or m^4^ N^−1^ s^−1^ being variously the units used) [113]. The permeability used in most of the cartilage literature derives from Darcy's law governing the flow of fluid through a porous medium. In simple form

$$Q=\frac{k_{0}}{\eta }\frac{\partial P}{\partial z},$$

where *Q* is the volumetric flow of fluid of viscosity, *η*, through a medium whose intrinsic permeability is *k_0_,* subject to a pressure gradient. The figure generally quoted for cartilage permeability is *k =* *k*_0_/*η* rather than the intrinsic permeability, *k*_0_, which has units of (length)^2^, is a property of the material and does not depend on the fluid. In this nomenclature and multiplying by the viscosity of water, McCutchen's value gives an intrinsic permeability of 4.1 × 10^−19^ m^2^, taking the viscosity of water to be 0.7 × 10^−3^ N s m^−2^ at 37°C. Other direct measurements on tissues from a variety of joints yielded values in the range 1–7 × 10^−13^ cm^3^ s g^−1^ (*k_0_* ∼ 10^−19^ m^2^) [114,115]. Permeability later became an essential part of biphasic theory developed by Mow *et al*. [116] and appears as one of three variables in the governing equations. Values were derived from fitting curves to experimental creep and stress relaxation experiments and, although not verified by independent means, led to predicted values in the region of 0.2 × 10^−14^ m^4^ N^−1^ s^−1^ [117], *k* = 2.2 ± 0.8 × 10^−15^ m^4^ N^−1^ s^−1^ [118], *k* = 1.42 × 10^−15^ m^4^ N^−1^ s^−1^ in patellar groove cartilage and *k* = 0.44 × 10^−15^ m^4^ N^−1^ s^−1^ in young bovine femoral condylar cartilage *in situ* [119]. Expressed as intrinsic permeability, by multiplying by the viscosity of water, these all come out in the range *k*_0_ ∼ 0.3–2.1 × 10^−18^ m^2^. Expressed as intrinsic permeabilities enables these values to be compared with other materials, notably various rocks. Limestone is considered a permeable rock with a porosity of approximately 20% and intrinsic permeability approximately 10^−16^ m^2^ [120]. By contrast, granite is considered to be impermeable and measurements of porosity and intrinsic permeability report figures for the porosity of approximately 0.5–4%, and an intrinsic permeability in the range 10^−17^−10^−19^ m^2^ [121,122]. Articular cartilage, then, has a permeability comparable with the least permeable granites; a most surprising result for a material that comprises about 75% water. Finally, if water flow is responsible only for the viscous element of the mechanical properties, restricted water flow due to inertia during rapid loading was predicted to result in increasing elasticity attributed to the ‘solid’ matrix. Instead, a recent study has indicated that the energetic coefficient of restitution of human cartilage, a measure of energy returned on unloading, decreases with drop-height during impact loading [112].

### 4.2. Swelling pressure

The high fixed charge density arising from the highly anionic GAG chains results in the attraction of counter-ions to maintain electrical neutrality. These counter-ions are mainly sodium (240–350 mM) with some potassium and calcium, in equilibrium with the synovial fluid. By treating cartilage as a semi-permeable membrane, the distribution of charges inside and outside the tissue can be calculated using the theory of Donnan equilibrium. The unequal distribution of particles [114,123] gives rise to an osmotic swelling pressure of about 0.2 MPa [26,124], amounting to slightly more than half of the pressure in the unloaded tissue [125,126]. The remainder comes from entropic effects [125]. In this model, if water is squeezed out of the tissue by loading then the internal ionic concentration increases thereby increasing the swelling pressure to resist the load. The low permeability, however, means that this process is slow compared with the timescale of physiological loading. During walking, the load rises to a maximum in about 100 ms and remains elevated during the stance phase of the gait cycle. This loaded period occupies about one-third of the cycle at 0.5–1 Hz typical of walking, and this fraction reduces as speed increases to just over 2 Hz when sprinting. This load–unload cycle is believed to enable the cartilage to re-imbibe fluid lost during loading. It has been shown, however, that this is less than the amount lost [127]. While this argument offers an intuitive approach to quasi-static loading, it becomes clear that the swelling pressure cannot be the mechanism supporting the load during such physiological processes, as it cannot rise fast enough due to the extremely low permeability. It may provide a balancing pressure at equilibrium, but most joint loading is far from equilibrium, and other mechanisms need to be invoked to support dynamic loads.

## 5. Mechanical properties

### 5.1. Conceptual models

Attempts to explain or describe the responses of articular cartilage to loads have adopted a variety of conceptual approaches. These include those based on mixture theory [116], electrostatic models [128], consolidation [129] and others which, although less quantitative, attempt to use the descriptions of structure in terms of composition and fibre organization [33,130]. The challenge is to explain the origins of the strongly time-dependent properties and the resistance to fracture necessary to support the range of forces applied during everyday life that mean we can perform activities from standing to jumping with impunity. The modulus of cartilage varies from less than 1 MPa during prolonged loading to about two orders of magnitude greater during impact [95,131,132]. Poisson's ratio and hysteresis increased with strain during impact loading [112], and we still lack a complete description of the mechanical properties in terms of compliance or stiffness spectra over a sufficiently wide range of frequencies commonly used to describe viscoelasticity of a material [133,134].

### 5.2. Biphasic theory and consolidation

The biphasic theory of Mow *et al*. [116] treats cartilage as separate incompressible fluid and solid phases. Arguably, this is still the most popular model despite early criticism that the theory did not properly fit the transient response arising from fluid transport [135] and that a development of the theory to triphasic [136] was shown to be thermodynamically impossible [137]. Oloyede and Broom believed that the deficiencies in biphasic theory arose because of the total separation between fluid and solid and that a consolidation model was more successful due to it considering the response of an integrated system [129]. Harrigan and Mann made a similar point that it was not possible to draw sharp distinctions between ‘solid’ and ‘fluid’ because they are mingled at a molecular level and phase boundaries do not exist [138]. They concluded that porosity cannot be meaningfully defined and the use of volume fractions to apportion stresses and balance masses is not physically defensible; they expressed surprise at the continued popularity of mixture theories.

Consolidation theories, adapting the theory developed for soils by Biot [7], do not necessarily draw such a clear distinction between solid and fluid phases [138]. Oloyede & Broom [139] pursued the question of whether consolidation, which was derived for materials which comprise predominantly a solid phase, could be applied successfully to a material such as cartilage with its high water content. Using a specially designed consolidometer, they measured what they termed ‘excess pore pressure’ and showed that it did behave as might be expected from classical consolidation theory. Pressure in the fluid, the excess pore pressure, rose on the application of load and decayed to zero with time. Unfortunately, despite suggesting the inappropriateness of distinguishing between solid and fluid phases they still concluded that this decay of pressure occurred as the ‘solid matrix’ began to bear the load. Such slow rates of loading compare well with equilibrium studies of swelling pressure described below, in which water extrusion generates increasing osmotic and entropic pressures. The rapid rise in pore pressure indicates that the fluid becomes pressurized during load imposition but gives little further insight into either static equilibrium or dynamic processes more typical of physiological loading.

### 5.3. Finite-element models

A rapid increase in computing power has led many to move towards a finite-element computational approach. Some of these models are highly sophisticated and contain multiple scalar, vector and tensor parameters (e.g. [140–142]). Most are based on biphasic theory with its implicit assumption of incompressibility and separability of the solid and liquid phases with all the limitations discussed above. While they might serve a descriptive purpose in models of joints, incorporating up to 13 material properties that can be varied [141] means that almost any mechanical property can be modelled but without necessarily increasing our understanding of how those properties arise from the macromolecular composition.

### 5.4. Polyelectrolyte

To understand the properties of cartilage from a molecular perspective, models have been developed and tested based on theories of polyelectrolytes, large highly charged macromolecular complexes [143]. A Poisson–Boltzmann-based molecular model was developed by Dean *et al*. [144] that considered CS as a cylindrical, charged rod with a uniform charge density. A series of papers addressing this model using atomic force microscopy and nanomechanics is beginning to show how the properties of cartilage depend on the macromolecular composition, and the ionic strength and pH of the molecular environment [23,143,145,146]. This approach is exciting because it is one of the first serious attempts to relate molecular composition to mechanical behaviour and begins to explore how much more we need to understand about the interactions between these components and with water if we are to design replacement tissues for implantation.

### 5.5. Fibre-reinforced composite

The previous models have focused largely on the polyelectrolyte components of the matrix and their relationship with water. These gels, however, are mechanically weak and the role of the stiff and strong collagen fibres is to provide reinforcing to this matrix to form essentially a new material; a fibre composite has emergent properties that are not easily predictable. The basic theories of fibre-reinforcing are well-established, especially for synthetic materials that are technologically important. Applying these ideas to biological materials is more difficult due to the nonlinear, time-dependent nature of the mechanical properties of the components. In addition, the properties of the composite depend not only those of the components but also on their organization and on how they interact. These interactions are governed by properties such as the size, shape and surface properties of the fibres. Collagen fibres grow from tapered ends [147,148], and this taper not only has biological consequences [149] but provides a sophisticated mechanism for maximizing stress transfer along the fibre length without producing a peak of stress in the centre of fibre that might lead to it being ruptured [150]. Collagen fibres have been shown to merge [151], which may provide one mechanism for transferring stress through a tissue, but calculations based on discontinuous fibre composite models indicated that strong cross-links were not necessary and that a large number of weak interactions, no stronger than hydrogen bonds, may be sufficient to fully stress a fibre [152]. Interactions between aggrecan and collagen fibres have recently been measured using atomic force microscopy and the adhesive forces shown to be non-specific [23]. The magnitude of these forces was calculated to be of the order of 0.3 pN per aggrecan monomer and about 9 pN per collagen fibril, of the same order as those estimated from a theoretical approach to stress transfer to collagen fibrils [152]. Adhesion was regulated by both GAG–GAG electrostatic repulsion and Ca^2+^-induced ion bridging [23].

Finally, the directions in which the fibres are oriented determine the directions in which the tissue can best withstand externally applied stresses [153], and the organization of fibres within the tissue then becomes crucial. The importance of this is that collagen fibre dimensions and orientation and their ability to interact with the matrix are all important in order to provide reinforcing to the weak proteoglycan gel. Fibre composition and surface modifications by attachment of other molecules, as described above, then become essential, and engineered tissue in which this does not happen is likely to be weak and mechanically inadequate. The challenge then is not simply to synthesize collagen, but collagen fibrils with the appropriate dimensions, shapes and surface properties to interact with the other matrix components.

## 6. Concluding remarks

The functions of many of the molecules comprising cartilage, often in significant molar amounts, are poorly understood. Going back to basics, however, may provide new directions and identify the key features that need to be investigated for successful repair or regeneration. Advances in neutron and X-ray scattering, especially the advent of high-brightness X-ray sources that are being used for high-resolution structural studies of cartilage may begin to advance our knowledge of tissue micromechanics [154]. Developments in atomic force microscopy, too, may help to uncover some of the hidden details. The picture that we present, however, is of a structure, rather than a material, organized by the cells and carefully constructed to contain water in a fibre-reinforced polyelectrolyte environment. The emphasis is not on restricting water flow but on holding and structuring water to generate a self-healing and resilient structure. Water is essential for the behaviour of the polyelectrolyte components by providing a medium with dielectric properties in which the charges can be distributed. It is also the only component that has mechanical properties in compression that are large enough to be modulated to generate the high stiffness of cartilage. The structure of cartilage is laid down in the embryo, and it may be there that we have to look for inspiration, as satisfactory repair of the adult tissue does not appear to happen naturally and implanted tissue faces many difficulties.

## Data Availability

This article has no additional data.
